# Biochanin A Ameliorates Arsenic-Induced Hepato- and Hematotoxicity in Rats

**DOI:** 10.3390/molecules21010069

**Published:** 2016-01-09

**Authors:** Abdulkadhar Mohamed Jalaludeen, Woo Tae Ha, Ran Lee, Jin Hoi Kim, Jeong Tae Do, Chankyu Park, Young Tae Heo, Won Young Lee, Hyuk Song

**Affiliations:** 1 Department of Animal Biotechnology, College of Animal Bioscience and Technology, Konkuk University, Seoul 143-701, Korea; bisnanojalal@gmail.com (A.M.J.); hows4u@nate.com (W.T.H.); ranran24@kku.ac.kr (R.L.); jhkim541@konkuk.ac.kr (J.H.K.); dojt@konkuk.ac.kr (J.T.D.); chankyu@konkuk.ac.kr (C.P.); urbangypsy@hanmail.net (Y.T.H.); 2 Division of Food Bioscience, College of Biomedical and Health Science, Konkuk University, Chung-ju 380-701, Korea; abseng@kku.ac.kr

**Keywords:** arsenic, biochanin A, lipid peroxidation, reactive oxygen species, selenium

## Abstract

Biochanin A (BCA) is a natural organic compound of the phytoestrogenic isoflavone class that has antioxidant and metal chelator properties in the presence of transition metal ions, however, its efficacy in animal models is still obscure. Therefore, the objective of this study was to investigate the protective effects of BCA against arsenic-induced hepatic injury and hematotoxicity in rats. The results suggest that arsenic intoxicated rats showed significantly higher levels of plasma hepatic markers than normal control rats. Furthermore, an increase in lipid peroxidation with depletion of reduced glutathione (GSH) and activities of superoxide dismutase (SOD) and catalase (CAT) occurred in the livers of rats exposed to arsenic. Administration of BCA (20 mg/kg·bw/day) and selenium (3 mg/kg·bw/day) resulted in a significant reversal of hepatic and oxidative stress markers in arsenic-intoxicated rats. A low dose of BCA (10 mg/kg·bw/day) did not show any preventive effect, while a high dose of BCA (40 mg/kg·bw/day) partially prevented all hepatotoxicity events. These biochemical perturbations were supported by histopathological observations of the liver. Our results suggest that administration of BCA (20 mg/kg·bw/day) attenuated the arsenic hepatotoxicity, a property that could contribute to the therapeutic approaches for chronic liver diseases.

## 1. Introduction

Arsenic, a ubiquitous metalloid, has become a major public health concern in some countries such as India, Bangladesh and Pakistan, *etc.* [1]. It has been utilized in the manufacture of wood preservatives, glass, semiconductors, dyestuffs, cigarettes, and herbicides [2]. Due to its increasing production and utilization in modern society, not only industrial workers but also the general population is exposed to the toxic effects of arsenic [3]. Drinking water and industrial pollution are the major routes of human exposure to inorganic arsenic [4]. Worldwide an estimated 200 million people, including a large number of children, are affected by arsenic exposure [5]. Numerous studies have reported associations between arsenic exposure and multiple adverse clinical manifestations [6].

Arsenic toxicity largely stems from the chemical form and physical state of the species involved. Trivalent inorganic arsenic is considered to be more toxic than pentavalent inorganic arsenic [7]. The trivalent arsenic toxicity could be mediated by its direct binding to vicinal thiols or biological ligands containing sulfur groups, and its participation in cellular redox reactions resulting in an increased generation of free radicals [8,9]. An epidemiological survey showed that chronic exposure to arsenic instigates hepatomegaly, hepatic fibrosis, and liver tumors in arsenicosis patients from endemic arsenic exposure areas [10,11].

A precise mechanism for arsenic-induced hepatotoxicity has yet to be elucidated; many reports have suggested that oxidative stress exacerbates hepatic toxic arsenic events [12,13]. Thus, it is believed that antioxidant administration may mitigate arsenic-induced toxicity. Isoflavones, a group of natural phytoestrogens, are present in plant foods and protect against heavy metal-induced oxidative stress-related diseases in experimental animals [14,15].

Biochanin A (5,7-dihydroxy-4-methoxyisoflavone, BCA) is a phytoestrogen, a natural biologically active isoflavonoid found in red clover [16], that has been studied extensively for its possible pharmacological activity, including anticancer, anti-inflammatory, neuroprotective, and anti-oxidant effects [17,18,19,20]. It was observed to protect against carbon tetrachloride-induced hepatotoxicity in rats [21]. In addition, BCA has better chelating and antioxidant effects than some other isoflavanoids using stoichiometry studies [22]. However, there are no studies on the effect of BCA on arsenic-induced hepatic damage and hematotoxicity in rats. Therefore, this study was designed to investigate the potential beneficial effects of BCA against arsenic-induced hepatotoxicity in rats. Moreover, selenium, which chelates arsenic, functions as an antagonist [23,24] and was selected as a reference compound to compare the efficacy of BCA in ameliorating arsenic hepatotoxicity in rats. We therefore selected selenium as a reference compound to determine the efficacy of BCA against arsenic hepatotoxicity in rats.

## 2. Results

### 2.1. General Characteristics

To determine the rescue effect of BCA on arsenic induced toxicity, we determined body weight, organ-body weight ratio, food intake, and water intake. There were no significant differences between the control and experimental groups (Table 1).

**Table 1:** General characteristics of normal control and experimental rats.

Groups	Body Weight (g)		Food Intake (g/rat/day )	Water Intake (mL/rat/day)	Liver Weight (g)	Liver-Body Weight Ratio (%)
	Initial	Final				
Normal control	203.33 ± 17.42	312.33 ± 27.41	14.66 ± 1.21	20.50 ± 1.84	21.16 ± 1.54	6.77 ± 0.12
Normal + Vehicle	202.00 ± 19.26	314.25 ± 28.46	14.83 ± 1.64	20.24 ± 1.92	21.06 ± 1.98	6.89 ± 0.06
Normal + BCA (40 mg/kg)	201.66 ± 18.22	315.24 ± 28.64	13.83 ± 0.98	20.74 ± 1.84	21.16 ± 1.12	6.74 ± 0.06
Arsenic (10 mg/kg)	205.33 ± 17.24	309.65 ± 26.48	13.16 ± 0.96	19.33 ± 1.74	21.86 ± 1.28	7.17 ± 0.48
Arsenic +Selenium (3 mg/kg)	203.45 ± 18.24	313.52 ± 28.46	13.00 ± 1.09	20.64 ± 1.84	22.13 ± 1.41	7.11 ± 0.14
Normal + BCA (10 mg/kg)	205.66 ± 18.42	309.84 ± 28.42	13.16 ± 1.21	20.68 ± 1.42	22.27 ± 1.58	7.22 ± 0.10
Normal + BCA (20 mg/kg)	208.15 ± 17.42	310.34 ± 24.52	13.50 ± 1.42	19.48 ± 1.75	22.12 ± 1.72	7.16 ± 0.12
Normal + BCA (40 mg/kg)	204.64 ± 15.26	310.54 ± 26.64	13.83 ± 1.16	19.95 ± 1.64	21.64 ± 1.84	6.99 ± 0.18

All values are expressed as means ± SD (*n* = 6).

### 2.2. Hepatic Markers

**Figure 1:**
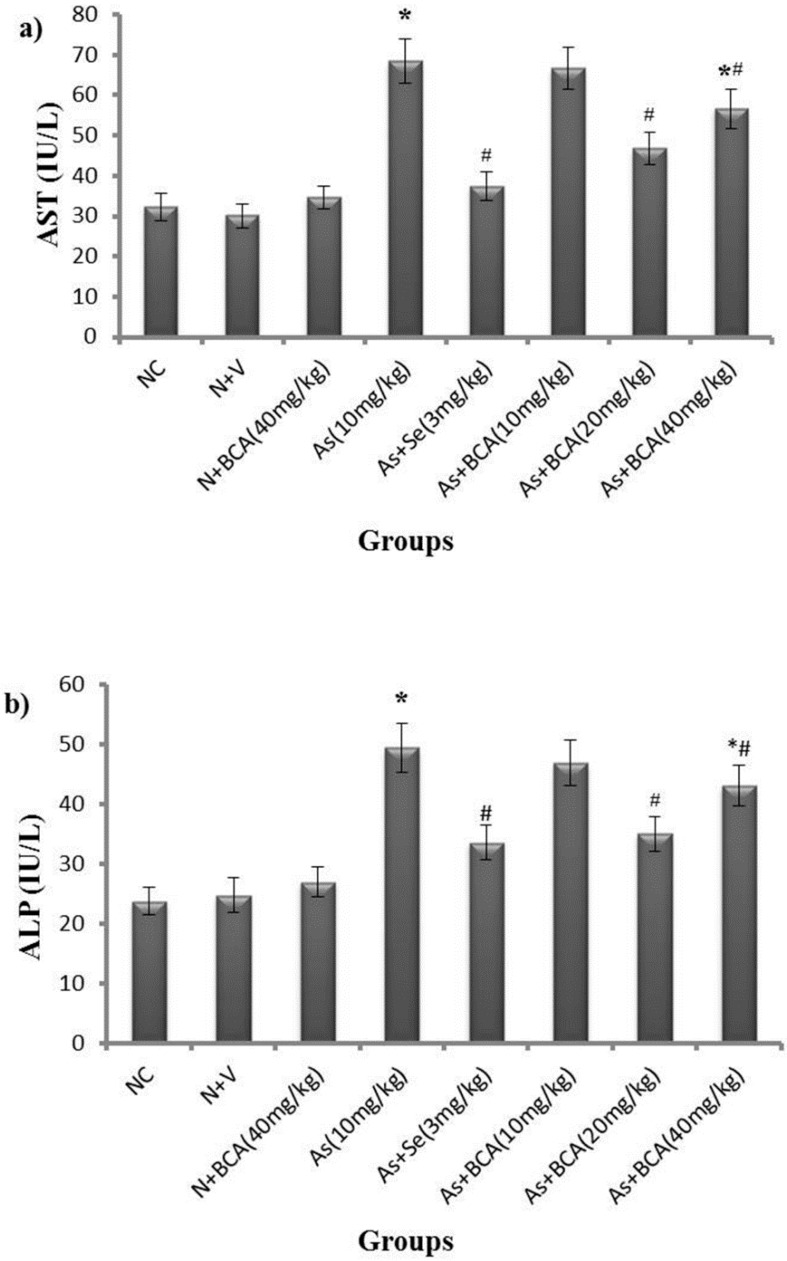
Differences between the activities of plasma AST (**a**) and ALT (**b**) in normal control and experimental rats. All values are expressed as means ± SD (*n* = 6). NC: Normal control, N: Normal, V: Vehicle, BCA: Biochanin A, As: Arsenic, Se: Selenium.* *p* < 0.05 compared to normal control rats. **^#^** *p* < 0.05 compared to arsenic-treated rats.

Next, we examined the important enzymes such as AST and ALT, which are actively involved in liver functions (Figure 1). The activities of AST and ALT were significantly higher in arsenic-intoxicated rats than in normal control rats. Administration of selenium and BCA (20 mg/kg·bw) protected the liver function against arsenic toxicity compared to the rats treated with arsenic alone. A high dose of BCA showed a somewhat beneficial effect, but rats treated with a low dose of BCA did not differ from arsenic-treated rats in AST or ALT activity. Supplementation with vehicle and BCA alone resulted in non-significant changes in these liver indices relative to the normal control rats. Taken together, the results indicate that BCA rescues the negative effects caused by arsenic.

### 2.3. Plasma Reduced Glutathione Level

**Figure 2:**
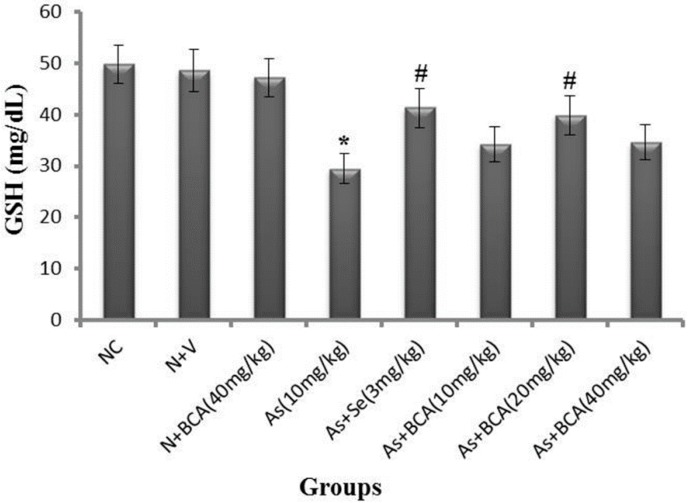
Differences between the level of plasma GSH in normal control and experimental rats. All values are expressed as means ± SD (*n* = 6). NC: Normal control, N: Normal, V: Vehicle, BCA: Biochanin A, As: Arsenic, Se: Selenium.* *p* < 0.05 compared to normal control rats. **^#^** *p* < 0.05 compared to arsenic-treated rats.

Plasma GSH is directly proportional to arsenic accumulation in tissues. Therefore, we measured the level of GSH in the plasma of normal control and experimental rats. As shown in Figure 2, arsenic-intoxicated rats showed significantly lower plasma GSH levels than normal control rats. Administration of selenium and BCA (20 mg/kg·bw) in addition to arsenic resulted in significantly higher plasma GSH than that observed in rats treated with arsenic alone. There was no difference in these parameters between BCA alone-or vehicle-treated rats and normal control rats.

### 2.4. Oxidative Stress Markers

Quantities related to oxidative stress in the liver of normal control and experimental rats are presented in Table 2. The levels of GSH and activities of SOD and CAT were significantly lower in the livers of arsenic-treated rats than in normal control rats, which indicate a disturbance in the delicate antioxidant/pro-oxidant balance. Moreover, arsenic intoxication had deleterious effects on the membrane integrity of the liver as evidenced by a significant increase in MDA levels. On the other hand, co-administration of selenium and BCA to arsenic-treated rats resulted in a recovery of the above mentioned parameters. A significant ameliorative effect of BCA with respect to the oxidative stress markers was observed at a dose of 20 mg/kg·bw; however, the other doses did not have a beneficial effect. Administration of vehicle and BCA alone had no effect on these variables in normal rats.

**Table 2:** Oxidative stress markers in the liver of normal control and experimental rats.

Groups	GSH μM/mg Tissue	SOD U/mg Tissue	CAT U/mg Tissue	MDA nmole/mg Tissue
Normal control	4.74 ± 0.26	3.47 ± 0.17	23.36 ± 0.26	0.78 ± 0.05
Normal + Vehicle	4.89 ± 0.86	3.69 ± 0.33	24.12 ± 0.02	0.81 ± 0.06
Normal + BCA (40 mg/kg)	4.95 ± 0.58	3.82 ± 0.10	25.04 ± 0.16	0.84 ± 0.07
Arsenic (10 mg/kg)	2.76 ± 0.10 *	2.06 ± 0.15 *	14.33 ± 0.06 *	1.56 ± 0.02 *
Arsenic + Selenium (3 mg/kg)	3.90 ± 0.24 ^#^	3.12 ± 0.05 ^#^	21.18 ± 0.15 ^#^	0.86 ± 0.02 ^#^
Arsenic + BCA (10 mg/kg)	2.98 ± 0.09	2.16 ± 0.04	16.59 ± 0.05	1.27 ± 0.03
Arsenic + BCA (20 mg/kg)	3.76 ± 0.23 ^#^	2.94 ± 0.10 ^#^	19.15 ± 0.04 ^#^	0.92 ± 0.02 ^#^
Arsenic + BCA (40 mg/kg)	3.37 ± 0.22	2.38 ± 0.05	18.44 ± 0.13 *^#^	0.94 ± 0.04 ^#^

All values are expressed as means ± SD (*n* = 6). * *p* < 0.05 compared to normal control rats. **^#^** *p* < 0.05 compared to arsenic-treated rats.

### 2.5. Phospholipids and Free Fatty Acid Levels

**Figure 3:**
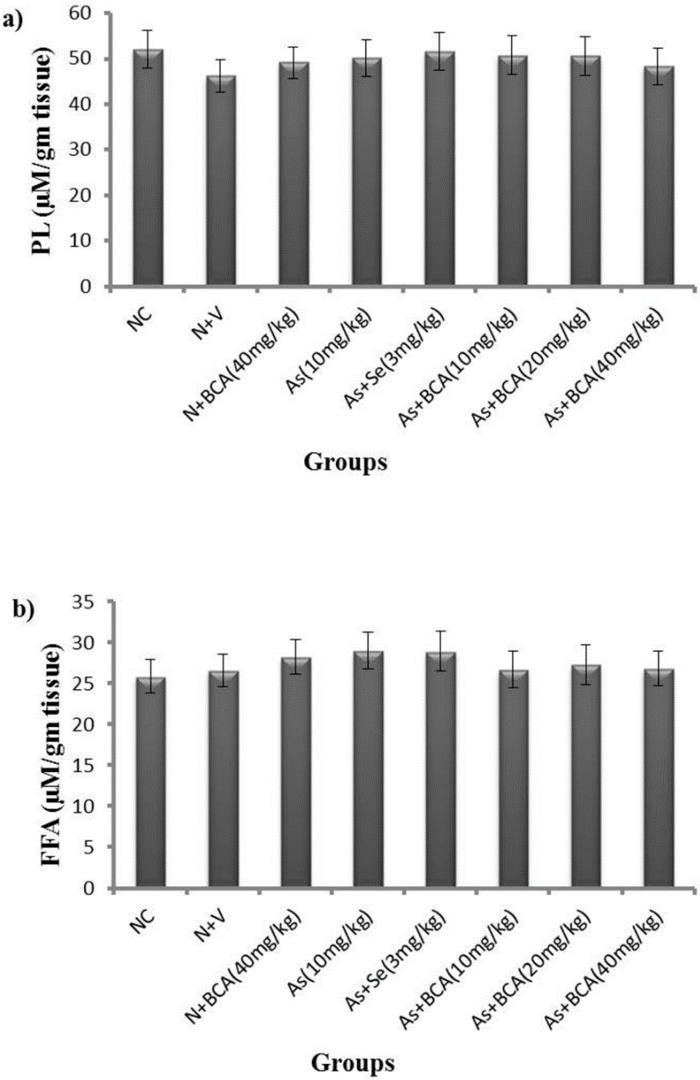
Differences between the levels of phospholipids (**a**) and free fatty acids (**b**) in livers of normal control and experimental rats. All values are expressed as means ± SD (*n* = 6). NC: Normal control; N: Normal; V: Vehicle; BCA: Biochanin A; As: Arsenic; Se: Selenium.

We investigated the role of phospholipids and free fatty acid levels arsenic induced hepatotoxicity by analyzing lipid metabolism in mice treated with arsenic with BCA or without BCA. The imbalance of phospholipid and free fatty acid levels are expected to cause injury. However arsenic did not significantly change the PL and FFA levels in the liver, as illustrated in Figure 3.

### 2.6. Hematological Indices

As depicted in Table 3 and Table 4, the arsenic-treated group showed significantly lower HCT, MCH, eosinophil, platelets, and MPV, and significantly higher WBC count, MCHC, neutrophils, and lymphocytes than the normal control rats. BCA and selenium supplementation mitigated the effect of arsenic treatment on the above mentioned variables, thus reducing hematotoxicity in arsenic-intoxicated rats. However, selenium-treated rats showed an increase in the levels of platelets, MPV, and WBC count compared to normal control rats. Only a low dose of BCA showed a significant ameliorative effect of arsenic treatment, especially with respect to the RBC indices. In contrast, vehicle-treated rats showed significantly higher RBC counts than normal control rats.

**Table 3:** RBC indices of normal control and experimental rats.

Groups	Normal Control	Normal + Vehicle	Normal + BCA (40 mg/kg)	Arsenic (10 mg/kg)	Arsenic + Selenium (3 mg/kg)	Arsenic + BCA (10 mg/kg)	Arsenic + BCA (20 mg/kg)	Arsenic + BCA (40 mg/kg)
RBC count (×10^6^ cells/L)	8.44 ± 0.32	7.3 ± 0.36 *	8.14 ± 0.38	8.14 ± 0.31	8.23 ± 0.31	8.67 ± 0.45	8.37 ± 0.52	8.27 ± 0.28
Hb (g/dL)	14.97 ± 1.12	14.51 ± 0.81	14.22 ± 0.73	14.5 ± 0.63	15.85 ± 1.20 ^#^	16.4 ± 0.52 ^#^	15.37 ± 0.84	15.17 ± 0.73
Hematocrit (%)	48.67 ± 3.51	47.15 ± 3.40	46.35 ± 3.82	44.52 ± 3.39 *	47.77 ± 3.37	50.77 ± 4.04 ^#^	47.4 ± 3.10	45.87 ± 3.24
MCV (fL)	57.57 ± 4.35	57.57 ± 4.30	56.45 ± 4.12	55.35 ± 4.06	58.07 ± 4.64	57.3 ± 4.13	56.65 ± 4.68	56.47 ± 4.60
MCH (pg)	23.70 ± 1.50	23.85 ± 1.25	23.37 ± 1.38	17.40 ± 0.84 *	23.97 ± 1.66 ^#^	24.27 ± 1.74 ^#^	24.32 ± 1.35 ^#^	23.87 ± 1.60 ^#^
MCHC(g/dL)	41.15 ± 3.98	41.45 ± 3.90	41.25 ± 3.47	43.85 ± 3.70 *	41.92 ± 3.37 ^#^	42.14 ± 3.20 ^#^	42.84 ± 3.21 ^#^	43.02 ± 3.86
CHCM (g/dL)	32.25 ± 2.36	32.22 ± 2.66	31.82 ± 2.35	32.35 ± 2.26	31.25 ± 2.31	31.20 ± 2.14	31.92 ± 2.28	32.00 ± 2.18
RDW (%)	13.50 ± 0.29	13.20 ± 0.69	13.00 ± 0.42	13.45 ± 0.31	13.25 ± 0.12	13.02 ± 0.18	13.02 ± 0.45	13.00 ± 0.20
HDW(g/dL)	2.81 ± 0.07	2.65 ± 0.03	2.69 ± 0.08	2.95 ± 0.09	2.87 ± 0.08	2.89 ± 0.06	2.88 ± 0.09	2.71 ± 0.02

All values are expressed as means ± SD (*n* = 6). * *p* < 0.05 compared to normal control rats. **^#^** *p* < 0.05 compared to arsenic-treated rats.

**Table 4:** WBC indices and platelets of normal control and experimental rats.

Groups	Normal Control	Normal + Vehicle	Normal + BCA (40 mg/kg)	Arsenic (10 mg/kg)	Arsenic + Selenium (3 mg/kg)	Arsenic + BCA (10 mg/kg)	Arsenic + BCA (20 mg/kg)	Arsenic + BCA (40 mg/kg)
WBC count (×10^6^ cells/L)	2.96 ± 0.12	3.10 ± 0.45	3.49 ± 0.64	6.27 ± 0.75 *	3.22 ± 0.37 ^#^	3.58 ± 0.28 ^#^	4.26 ± 0.31 ^#^	4.05 ± 0.31 ^#^
Monocytes (%)	2.25 ± 0.36	2.62 ± 0.31	2.50 ± 0.32	1.90 ± 0.59	2.32 ± 0.15	2.05 ± 0.40	2.25 ± 0.23	1.85 ± 0.44
Easinophil(%)	2.22 ± 0.17	2.17 ± 0.29	2.25 ± 0.62	1.30 ± 0.29 *	1.98 ± 0.43 ^#^	0.82 ± 0.12	1.00 ± 0.08	1.22 ± 0.34
Basophil (%)	0.12 ± 0.02	0.10 ± 0.08	0.13 ± 0.08	0.10 ± 0.01	0.14 ± 0.09	0.12 ± 0.09	0.15 ± 0.05	0.10 ± 0.01
Leucocytes (%)	0.12 ± 0.05	0.20 ± 0.03	0.17 ± 0.05	0.22 ± 0.05	0.15 ± 0.05	0.12 ± 0.05	0.15 ± 0.05	0.12 ± 0.09
Neutrophil (%)	16.60 ± 0.98	13.52 ± 0.85	15.75 ± 1.24	26.1 ± 1.21 *	18.77 ± 1.63 ^#^	23.27 ± 1.37	19.22 ± 1.28 ^#^	21.05 ± 1.99
Lymphocytes (%)	69.42 ± 4.58	81.55 ± 6.51 *	77.75 ± 5.97	80.32 ± 6.83 *	74.90 ± 5.50	80.87 ± 6.78	77.52 ± 5.42	75..62 ± 6.24
Platelets (×10^6^ cells/L)	943 ± 83.44	917.25 ± 76.50	941.25 ± 75.56	616.50 ± 59.37 *	1034.8 ± 98.77 ^#^	721.50 ± 66.92	936.50 ± 82.73 ^#^	864.50 ± 76.41 ^#^
MPV(fL)	10.12 ± 0.94	11.63 ± 0.94	10.5 ± 0.84	6.78 ± 0.83 *	11.55 ± 0.80 ^#^	12.37 ± 0.79 ^#^	10.95 ± 0.67 ^#^	11.50 ± 0.45 ^#^

All values are expressed as means ± SD (*n* = 6). * *p* < 0.05 compared to normal control rats. **^#^** *p* < 0.05 compared to arsenic-treated rats.

### 2.7. Histopathological Observations in the Liver

**Figure 4:**
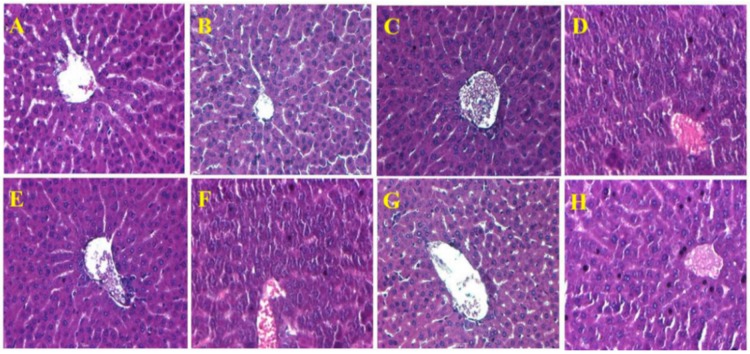
Hematoxylin and eosin-stained sections of rat livers (40×): (**A**) Normal control rat liver: Normal liver architecture; (**B**) Vehicle alone-treated rat liver: Normal liver architecture; (**C**) Biochanin A (40 mg/kg)—treated rat liver: Normal appearance of liver cells; (**D**) Arsenic (10 mg/kg)—treated rat liver: Blurred appearance, absence of intact nuclei and RBC congestion; (**E**) Arsenic + Selenium (3 mg/kg)—treated rat liver: Normal hepatocytes; (**F**) Arsenic + Biochanin A (10 mg/kg)—treated rat liver: Blurred appearance, absence of intact nuclei and congestion of RBC; (**G**) Arsenic + Biochanin A (20 mg/kg)—treated rat liver: Normal architecture of liver with disarrangement of radial pattern of hepatocytes; (**H**) Arsenic + Biochanin A (40 mg/kg)—treated rat liver: Disarrangement in radial pattern of hepatocytes and sinusoidal space.

Figure 4 shows a representation of the hepatic architecture of normal control and experimental rats.

Normal control rats showed regular histological structure with a characteristic pattern of hepatocytes arranged into hepatic cords running radiantly from the central vein. In contrast, arsenic-exposed rats revealed pathological alterations such as the congestion of RBC, absence of intact nuclei, and a blurred appearance, which are all involved in the initiation of fibrosis. Furthermore, no histological alterations were observed in the livers of vehicle- and BCA alone-treated rats when compared to the normal control rats; however, arsenic combined with selenium and BCA (20 mg/kg·bw) supplementation showed prominent recovery in the form of reduced RBC congestion, blurred appearance, and collapse of sinusoidal spaces. No positive sign and sinusoidal space disturbance were observed in the low and high dose of BCA, respectively.

## 3. Discussion

Phyto-antioxidants are gradually replacing conventional chelating agents in combating arsenic toxicity. BCA, a natural isoflavonoid, has been observed to have chelating and antioxidant properties in the presence of transition metal ions due to the orientation of its functional groups [25]. It is well established that arsenic induces alterations in the function of hepatic machinery in both experimental and human studies [26,27]. Hence, in this study, the therapeutic potential of BCA was compared with selenium on arsenic hepatotoxicity and examined in rats with reference to hepatic markers, oxidative stress parameters, lipid profiles, hematological variables, and liver histology.

The activities of AST and ALT are sensitive indices of hepatic injury. Numerous studies have reported that elevated activities of AST and ALT in the plasma of arsenic-exposed rats are mainly attributed to cellular leakage and loss of functional integrity of hepatic membrane architecture [28,29]. Consistent with previous studies, we observed significantly higher levels of AST and ALT in rats exposed to arsenic than in control rats, probably resulting from hepatocyte membrane damage due to arsenic intoxication. Administration of BCA and selenium attenuated arsenic-induced hepatotoxicity, as shown by the decreased levels of hepatic markers; stabilization of functional activity of the cell membrane might be attributed to their membrane protective effects [30,31].

The potential toxicity of arsenic can therefore be attributed to its inhibitory effects on the antioxidant defense system. GSH is a tripeptide non-enzymatic antioxidant required for the regeneration of other antioxidants like vitamin C and vitamin E to their active forms. Moreover, it is believed to be a potential intracellular reductant for arsenic methylation and transport. Thus, the reduction of GSH facilitates arsenic accumulation in the liver and causes oxidative stress [32]. In the present research, the level of GSH was remarkably increased in the plasma and liver of arsenic-treated rats. This is probably due to the collective consequences of free radical generation, its electron donating ability, and its affinity to arsenic.

On the other hand, SOD and CAT are enzymatic antioxidants that function mutually to eliminate free radicals produced during arsenic exposure [33]. SOD catalyzes the reduction of superoxide radical into hydrogen peroxide, while CAT reduces hydrogen peroxide into water and oxygen [34]. Available data have suggested that diminished activity of SOD and CAT in arsenic-exposed rats was due to the enhanced production of superoxide radical anions and insufficient supply of NADPH, respectively [35,36]. Our data also showed that the activities of SOD and CAT were significantly increased in the livers of rats treated with arsenic alone. These results revealed arsenic toxicity targets antioxidant enzymes that offer protection against oxidative stress.

Changes in antioxidant parameters and peroxidation of membrane lipids under arsenic exposure have been documented in animal models [37,38]. Moreover, the ROS generated by arsenic are known to destabilize cell membranes via lipid peroxidation, which is a basic cellular deteriorating process in the liver [2]. Available data have suggested that profound ROS generation and enhanced lipid peroxidation are dual effects of oxidative stress in the pathogenesis of arsenic hepatotoxicity [39]. MDA is a stable metabolite of the free radical-mediated lipid peroxidation cascade and is therefore a reliable index of lipid peroxidation [40]. In the present investigation, arsenic administration resulted in a significant increase in the MDA level, which confirms the oxidative damage of the liver.

Administration of BCA attenuated the elevation of lipid peroxidation and decreased the antioxidant parameters in the liver of arsenic-exposed rats. This might be due to the lipophilic nature of BCA, which favors its passage through the membrane and can accumulate in the lipid bilayer, thereby protecting the radical attack and maintaining the normal membrane physiology. BCA has the ability to scavenge free radicals in the presence of transition metal ions based on *in vitro* assays [25]. The maintenance of GSH, SOD, and CAT in the liver also provides information on the appropriate free radical scavenging properties of BCA against arsenic toxicity. Our data suggest that BCA has the capacity to counteract arsenic-induced oxidative damage in the liver; this could be due to its antioxidant nature, including its free radical scavenging and membrane stabilizing properties.

Arsenic has strong sulfhydryl affinity; therefore, it can alter lipid levels because most lipid metabolizing enzymes contain sulfhydryl groups. Furthermore, oxidative injury caused by arsenic is linked to alterations in the lipid homeostasis in the liver [41]. However, few studies have reported differences between rat strains in lipid metabolism after arsenic exposure. It did not observe a significant change in the lipid profile of rats following the ingestion of arsenic (50 μg/L) in drinking water for 260 days [42]. Our experiment showed that PL and FFA levels in the liver did not differ significantly between the experimental groups. This might be explained by the interaction of arsenic with GSH, rather than lipid metabolizing enzymes and insufficient supply of NADPH.

Oxidative stress markers are highly correlated with histological changes in the liver. Histopathological observations after arsenic administration showed congestion of WBC, initiation of fibrosis, and disarrangement of hepatic structure, and these data were consistent with previous reports [43]. Treatment with selenium and BCA decreased congestion of WBC, initiation of fibrosis, and disarrangement of hepatic structure. This could indicate improved hepatocyte function or reduced damage to cells in the presence of BCA and selenium.

Hematological parameters have been utilized to analyze the toxicity of environmental toxins and drugs in humans and animals. Recently, it has reported that most of the hematological parameters are not affected by arsenic exposure, except WBC and PLT count [44]. Here, arsenic exposure led to increases in the WBC count, neutrophils, and lymphocytes, decreases in eosinophil, and no alterations in monocytes, basophils, and leucocytes compared to normal control rats. Our erythrogram results indicated that arsenic exposure decreases the HCT, MCV, and MCH, but increases the MCHC, with normal RBC, HG, RDW, CHCM, and HDW. In addition, arsenic caused thrombocytopenia, as indicated by a decreased level of platelets and MPV compared with normal control rats. These alterations in the hematopoietic system could be the result of the normal detoxification of arsenic and its metabolites in the liver. Our findings were in partial agreement with previous reports [45,46]. Supplementation of BCA (20 mg/kg·bw) and selenium mitigated the effect of the above mentioned variables, thus reducing hematotoxicity in arsenic-intoxicated rats. These results might be attributed to the free radical scavenging activity of BCA. Altogether, the lower dose (20 mg/kg) of BCA showed the strongest effects although the higher dose (40 mg/kg) did statistically significant differences. This might be attributed to the metabolic nature of BCA. BCA can be metabolized initially into genistein. If excess BCA is available, it is metabolized into both genestin and daidzein. The daidzein showed poor antioxidant property than other isoflavones and also induced oxidative stress by generating free radicals. We suggested that daidzein might be the reason for the efficacy of high dose of BCA is lower than medium dose of BCA.

## 4. Materials and Methods

### 4.1. Animals

Adult male Sprague-Dawley rats (4 weeks old) were obtained from the Central Lab (Seoul, Korea) and housed in solid bottom polypropylene cages under standard environmental conditions (12 h light/dark cycle; 50% ± 10% humidity; temperature 23 ± 2 °C). Commercial pellet diet and water were fed ad libitum. The care and treatment of rats were in accordance with the guidelines established by the Korean National Institute of Health at the Korean Academy of Medical Sciences and were approved by the Institutional Animal Care and Use Committee (IACUC) of Konkuk University (KU14308).

### 4.2. Chemicals and Reagents

BCA, selenium, and sodium meta-arsenite were purchased from Sigma Chemical Co. (St. Louis, MO, USA). Aspartate aminotransferase and alanine aminotransferase kits were obtained from Asan Pharmaceuticals (Seoul, Korea). Glutathione, free fatty acid (FFA), phospholipids (PL), superoxide dismutase (SOD), and catalase (CAT) assay kits were procured from BioAssay Systems (Hayward, CA, USA). A malondialdehyde (MDA) assay kit was purchased from BioVision (Hayward, CA, USA). The rest of the chemicals utilized in the present study were obtained from local firms in South Korea and were of analytical grade.

### 4.3. Preparation and Administration of BCA, Selenium and Arsenic

A daily oral intake of BCA (5–50 mg/kg·bw) can reach plasma concentrations lower than or equal to ≤1 μM in rats [47]. In fact, the maximum plasma concentration of any isoflavone hardly exceeds ≤1 μM following dietary intake [48]. Therefore, we planned to examine the effect of BCA at a physiologically relevant concentration against arsenic toxicity in rats. BCA was suspended in 0.5% carboxymethylcellulose (CMC) and administered intragastrically at doses of 10, 20, and 40 mg/kg·bw after a hour exposure to arsenic for the duration of the experimental period. The arsenic and selenium doses used in the present study were based on previous reports [4,49]. Arsenic as sodium meta-arsenite was dissolved in water and administered intragastrically (10 mg/kg·bw/day) for 6 weeks. Selenium as sodium selenite was dissolved in water and treated orally (3 mg/kg·bw/day) following the same procedure as BCA.

### 4.4. Experimental Time Line

After a one week of acclimatization, rats were randomly divided into eight groups (six rats per group). Group I included normal control rats, Group II included normal rats orally administered 0.5% CMC (vehicle treated), Group III included normal rats orally administered BCA (40 mg/kg·bw/day),Group IV included normal rats that received arsenic as sodium meta-arsenite orally (10 mg/kg·bw/day), Group V included normal rats that received arsenic with co-administration of selenium orally (3 mg/kg·bw/day), and Groups VI–VIII included normal rats that received arsenic with co-administration of BCA orally at various doses (10, 20, and 40 mg/kg·bw/day) for 6 weeks. During the experimental period, food and water consumption were measured daily and rats were weighed every week.

### 4.5. Collection of Samples

At the end of experimental period, all rats were deprived of food overnight and were anesthetized with 4% avertin. Blood samples were collected in EDTA tubes through cardiac puncture from the left ventricle and centrifuged for 10 min at 1500× *g*. The clear plasma obtained was stored at −20 °C for various biochemical assays. The liver was also removed surgically after carefully opening the abdominal cavity. The collected liver tissues were washed with ice-cold saline and stored in sterile plastic vials at −20 °C for further analysis. For histological studies, hepatic tissues were placed immediately in the Bouin’s solution. Each set of experiments was repeated at least three times.

### 4.6. Measurement of Body Weight and Organ Body Weight Ratio

Body weights of rats before treatment and at the end of the treatment period were measured for all groups. The liver weight of every rat was measured after sacrifice and the organ weight (liver) to body weight ratio was determined using the body weight of each animal.

### 4.7. Assessment of Hepatic Damage

The activities of serum aspartate aminotransferase (AST) and alanine aminotransferase (ALT) were measured using commercially available diagnostic kits according to the manufacturer’s instructions. Their activity was detected using an ultraviolet/visible scanning spectrophotometer.

### 4.8. Estimation of Reduced Glutathione

Reduced glutathione (GSH) was measured in plasma and tissue homogenates with the QuantiChrom Glutathione Assay Kit (BioAssay Systems). In this assay, 5,5′-dithiobis (2-nitrobenzoic acid) reacts with reduced glutathione to form a yellow product. The optical density (OD) was measured at 412 nm by UV-Visible spectrophotometer, and is directly proportional to the glutathione concentration in the sample. The assay was conducted according to the manufacturer’s instruction.

### 4.9. Estimation of Lipid Peroxidation

Lipid peroxidation was estimated in the tissue homogenates by a colorimetric assay kit (BioVision, Milpitas, CA, USA). In brief, MDA in the samples is reacted with thiobarbituric acid to generate the MDA-TBA adduct, which can be easily quantified colorimetrically by UV-Visible spectrophotometer (OD 532 nm) as per the manufacturer’s instructions.

### 4.10. Assay of Antioxidant Enzymes

CAT and SOD activities were estimated in tissue homogenates using commercially available diagnostic kits (BioAssay Systems) by a colorimetric method following the manufacturer’s instructions. The changes in color intensity at 440 nm and 570 nm are directly proportional to the activities of SOD and CAT, respectively, in the samples.

### 4.11. Evaluation of Lipid Profiles

FFA and PL in the liver homogenate were quantified with the EnzyChrom Free Fatty Acids and Phospholipids Assay Kits (BioAssay Systems) respectively, according to the manufacturer’s instructions.

### 4.12. Clinical Hematological Variables

The collected whole blood samples were inverted several times in an EDTA-coated tube to prevent coagulation. The WBC indices, RBC indices, and platelets were analyzed immediately by an ADVIA 2120 hematology system (Siemens, Munich, Germany), according to the manufacturer’s instructions.

### 4.13. Morphological Studies of the Liver

For a qualitative liver histology analysis, the tissue samples were fixed for 48 h in Bouin’s solution, dehydrated by passing through a mixture of ethyl alcohol and water, cleaned in xylene, and embedded in paraffin. Sections of the liver tissues (5–6 μm thick) were prepared using a rotary microtome and stained with hematoxylin and eosin dye, which was mounted in a neutral deparaffinized xylene medium for microscopy observations at 40× magnification.

### 4.14. Statistical Analysis

Data are presented as means ± SD and evaluated by one-way analysis of variance (ANOVA) implemented in SPSS Version 13.0 (SPSS, Cary, NC, USA). The individual comparisons were assessed using Duncan’s multiple range test (DMRT). Differences were considered statistically significant when *p* < 0.05.

## 5. Conclusions

BCA is an *O*-methylated isoflavone, which is found in red clover, soy, alfalfa sprouts, peanuts and chickpea and it has an important role as an antioxidant in our diet. BCA has a broad spectrum of biological benefits, including anti-inflammatory, anti-oxidative and antineoplastic effects. Antioxidants can control several actions in cells through various signaling pathways. To understand their mechanisms of action it is necessary to evaluate these potent biomolecules in animal models. The results from this study suggest that arsenic intoxicated rats showed significantly higher levels of plasma hepatic markers than normal control rats. Furthermore, the lower level of oxidative stress markers such as GSH, SOD, CAT and increased lipid peroxidation indicate that arsenic treatment clearly induce toxicity in rats, whereas administration of BCA (20 mg/kg·bw/day) and selenium (3 mg/kg·bw/day) resulted in a significant reversal of hepatic and oxidative stress biomarkers in arsenic-intoxicated rats. Further, the results from our studies indicated that administration of BCA has protective effects very similar to those of selenium, especially at a higher dose of 20 mg/kg whereas the low dose of BCA (10 mg/kg·bw) has no significant impact except on the WBC indices; similarly, the effect of the higher dose of BCA (40 mg/kg·bw) did not differ significantly from that of the medium dose 20 mg/kg·bw BCA. This study suggests that 20 mg/kg·bw BCA could be an optimal dose to exert an antioxidant effect as a metal chelator in animal models. However, further studies are essential to elucidate the specific mechanisms by which BCA protects against arsenic-induced hepatic oxidative injury in rats.
